# Unmasking Additional Mutations: A Single Institution Study in Myeloproliferative Neoplasms

**DOI:** 10.3390/cancers18152458

**Published:** 2026-07-31

**Authors:** Parastou Tizro, Eric Vail, Manoj Sapkota, Matthew G. Gayhart, Celeste C. Eno

**Affiliations:** 1 Department of Pathology and Laboratory Medicine, City of Hope Comprehensive Cancer Center, Duarte, CA 91010, USA; ptizro@coh.org; 2 Department of Pathology and Laboratory Medicine, Cedars-Sinai Medical Center, 8723 Alden Drive, SSB-362, Los Angeles, CA 90048, USA; eric.vail@cshs.org (E.V.); matthew.gayhart@cshs.org (M.G.G.); 3 Department of Horticulture, University of Kentucky, Lexington, KY 40546, USA; manoj.sapkota@uky.edu

**Keywords:** MPN, *CALR*, *JAK2*, *MPL*, mutation, HMR, prognosis

## Abstract

Background/Objectives: Myeloproliferative neoplasms (MPNs) are chronic myeloid malignancies characterized by substantial genetic heterogeneity. Although routine molecular evaluation focuses on canonical driver alterations (*BCR::ABL1*, *JAK2*, *CALR*, and *MPL*), additional somatic mutations have important diagnostic and prognostic implications. Methods: We retrospectively analyzed 1209 consecutive patients who underwent peripheral blood next-generation sequencing (NGS) for suspected or established MPNs using a comprehensive 75-gene DNA/RNA panel. Results: Canonical driver alterations were identified in 141 patients, while 1068 were driver-negative. Retrospective analysis demonstrated that 251 patients (21%) harbored at least one additional pathogenic or likely pathogenic mutation that was not routinely reported. Among driver-positive patients, 57 of 141 (40%) carried clinically relevant co-mutations, including high-molecular-risk genes such as *ASXL1*, *TP53*, *SRSF2*, *SF3B1*, *RUNX1*, *U2AF1*, and *IDH2*. Furthermore, 194 of 1068 (18%) driver-negative patients harbored additional pathogenic mutations, several of which supported clonal hematopoiesis or alternative myeloid neoplasms. Peripheral blood and bone marrow molecular findings demonstrated high concordance (R^2^ = 0.87). Conclusions: These findings indicate that comprehensive NGS performed at the time of initial evaluation provides clinically relevant genomic information beyond canonical driver mutations, improves disease classification and risk stratification, and may facilitate earlier diagnosis and personalized management of patients with suspected MPNs.

## Simple Summary

Myeloproliferative neoplasms are chronic myeloid neoplasms that are usually diagnosed by testing for a small number of well-known genetic changes. However, many patients carry additional mutations that may influence disease behavior, risk of progression, and response to treatment. In this study, we analyzed more than 1200 patients who underwent molecular testing for suspected myeloproliferative neoplasms using a broad next-generation sequencing panel. We found that many patients had clinically relevant mutations beyond those routinely reported, including mutations associated with higher-risk disease and poorer outcomes. These additional findings were present not only in patients with established myeloproliferative neoplasms but also in some patients whose routine testing was reported as negative. Our results suggest that comprehensive genetic profiling at diagnosis can improve disease classification, risk assessment, and patient management. Broader use of genomic testing may support more personalized care and help identify individuals who could benefit from closer monitoring or earlier intervention.

## 1. Introduction

The abnormal proliferation of one or more terminally differentiated myeloid cell lines in the peripheral blood results in a heterogeneous group of disorders known as myeloproliferative neoplasms (MPNs). The World Health Organization classifies MPNs into eight subtypes. Among these, chronic myeloid leukemia (CML) is the most recognized, characterized by the distinctive *BCR::ABL1* rearrangement. There are also *BCR::ABL1*-negative MPNs, with major subgroups including polycythemia vera (PV), essential thrombocythemia (ET), and primary myelofibrosis (PMF) [1,2]. Classical Philadelphia chromosome-negative myeloproliferative neoplasms are uncommon clonal hematologic malignancies with a relatively low annual incidence; however, their prevalence continues to increase because of improved patient survival and earlier diagnosis [3,4].

The pathogenesis of MPNs is driven by acquired genetic alterations that promote clonal expansion of hematopoietic stem cells. In CML, this process is initiated by the *BCR::ABL1* fusion, whereas the pathogenesis of Philadelphia chromosome-negative MPNs is characterized by constitutive activation of the JAK-STAT signaling pathway. Canonical driver mutations in *JAK2*, *CALR*, and *MPL* account for the majority of classical Philadelphia chromosome-negative MPNs. However, disease phenotype, progression, and clinical heterogeneity are further influenced by additional somatic mutations affecting epigenetic regulation, RNA splicing, transcriptional control, and signal transduction [1,2,5].

MPNs exhibit a range of overlapping phenotypes, making accurate differentiation crucial for effective treatment. In over 95% of MPN cases, one of three genes—*JAK2*, *CALR*, or MPL—exclusively drives the disease phenotype [2]. *JAK2* p.V617F is the most common driver alteration in MPNs, occurring in >95% of patients with PV and approximately 50–60% of those with ET or PMF. In patients with *JAK2* p.V617F-negative PV, activating JAK2 exon 12 mutations (typically small in-frame insertions or deletions affecting the pseudokinase domain) are identified in the majority of cases. *CALR* exon 9 frameshift mutations are detected in approximately 25–35% of patients with ET and PMF and are not observed in PV. The two most common *CALR* alterations are the 52-bp deletion (type 1) and the 5-bp insertion (type 2), both of which generate a novel C-terminal protein sequence. Activating *MPL* mutations are identified in approximately 5–10% of patients with ET and PMF, most commonly involving codon W515, and are typically associated with thrombocytosis and/or bone marrow fibrosis in the absence of erythrocytosis [1,2].

The initial assessment of patients with suspected MPNs frequently begins with clinical evaluation, complete blood count abnormalities, and peripheral blood molecular testing. Although bone marrow examination remains essential for diagnosis and distinguishing several MPN subtypes, peripheral blood molecular testing is often favored because of its accessibility, lower cost, and minimally invasive nature [6]. In selected patients with unequivocal polycythemia vera characterized by sustained erythrocytosis, a *JAK2* mutation, and supportive clinical and laboratory findings, a bone marrow biopsy may not be necessary to establish the diagnosis. However, bone marrow evaluation continues to provide important prognostic and morphologic information [7].

In current clinical practice, molecular analysis begins with testing for *BCR::ABL1* using quantitative polymerase chain reaction (PCR) or fluorescent in situ hybridization (FISH). If the result is negative or suggestive on a non-CML MPN, the next step is typically to test by allele-specific oligonucleotide PCR for the *JAK2* V617F mutation, as it is the most common driver mutation, occurring in over 90% of patients with PV. In patients presenting with sustained erythrocytosis suggestive of polycythemia vera, analysis for *JAK2* exon 12 mutations is recommended, whereas *CALR* and *MPL* mutation testing is primarily indicated in patients presenting with thrombocytosis or suspected essential thrombocythemia or primary myelofibrosis, as these are found in approximately 60% of patients with ET or PMF [4,8].

The widespread implementation of next-generation sequencing (NGS) has fundamentally transformed the molecular evaluation of myeloid neoplasms by enabling simultaneous analysis of numerous clinically relevant genes. Recent updates to both the WHO Classification and the International Consensus Classification (ICC) emphasize integrating molecular findings with morphologic and clinical features to improve diagnostic accuracy and disease classification [1,2]. Comprehensive genomic profiling is particularly valuable in patients with atypical clinical presentations, borderline morphologic findings, or triple-negative disease, in whom molecular evidence of clonality may be difficult to establish using conventional testing alone. In addition to identifying canonical driver mutations, broader NGS panels detect mutations with diagnostic, prognostic, and potential therapeutic significance in a single assay, thereby reducing the need for sequential testing and shortening diagnostic turnaround time [1,2,5].

Several studies have demonstrated that, beyond the canonical driver mutations, a diverse spectrum of co-occurring somatic mutations contributes to substantial interpatient variability in the genetic and clonal landscape of myeloproliferative neoplasms. These additional mutations, including *ASXL1*, *EZH2*, *SRSF2*, *IDH1/2*, *U2AF1*, *TP53*, and *RUNX1*, not only contribute to phenotypic heterogeneity but are also associated with disease progression, leukemic transformation, inferior survival, and response to therapy [9,10,11,12,13,14]. Furthermore, both the variant allele frequency and the temporal order in which mutations are acquired can influence disease phenotype and clinical behavior [15,16,17]. Consequently, several of these molecular alterations have been incorporated into contemporary prognostic models, including the Mutation-Enhanced International Prognostic Scoring System (MIPSS70+ version 2.0) [18] and the Genetically Inspired Prognostic Scoring System (GIPSS) [19], underscoring their prognostic significance beyond the canonical driver mutations. Collectively, these advances highlight the growing role of comprehensive genomic profiling in precision medicine and support the consideration of broader NGS testing early in the routine diagnostic evaluation of patients with suspected MPNs [18,19]. NGS panel testing is now performed for risk assessment in transplant-eligible patients with myelofibrosis, but it has not yet been fully incorporated into routine clinical practice [20].

During the pandemic, our institution suspended single-gene testing and transitioned to using an NGS panel, though we only clinically report on four genes and *BCR::ABL1* fusion. To enhance patient care, we conducted a single institution study to analyze the unreported data, aiming to determine if utilizing the full panel could lead to more precise and timely diagnoses, as well as providing prognostic insight.

## 2. Method

### 2.1. Study Population and Design

This retrospective study was approved by the Cedars-Sinai Medical Center Institutional Review Board (STUDY00003102) in November 2023 and was conducted in accordance with the ethical principles of the Declaration of Helsinki. The study population included all consecutive patients referred from the hematology clinic for molecular evaluation because of suspected or previously established myeloproliferative neoplasms (MPNs) over a 20-month period. The clinical indications, including patients undergoing initial diagnostic evaluation and those with a prior diagnosis of MPN (Supplementary Table S1). Following standard clinical evaluation, including complete blood count, peripheral blood smear review, and appropriate laboratory investigations for unexplained cytosis, cytopenia(s), or combined cytopenia/cytosis, peripheral blood specimens were submitted for comprehensive molecular testing. Clinical data, laboratory findings, bone marrow pathology, cytogenetic studies, and follow-up information were retrospectively reviewed. Patient outcomes were collected through the end of the study period or the date of last clinical follow-up, and subsequent diagnoses of hematologic neoplasms established on peripheral blood or bone marrow examination were recorded. The study design did not allow for the evaluation of long-term clinical outcomes such as hematological transformation or thrombotic complications in addition to the diagnostic outcome.

### 2.2. Next-Generation Sequencing

Clinical comprehensive molecular profiling was performed on submitted specimens using the Ion Torrent Genexus Integrated Sequencer (Genexus v2 platform; Thermo Fisher Scientific, Waltham, MA, USA). Library preparation, sequencing, and primary data analysis were performed using the fully automated Genexus Integrated Sequencer.

Targeted sequencing was performed using the Oncomine Myeloid Assay (Thermo Fisher Scientific), an amplicon-based next-generation sequencing panel designed for the evaluation of myeloid malignancies. The assay interrogates hotspot mutations, insertions/deletions, and selected coding regions across 45 genes recurrently altered in myeloid neoplasms while simultaneously assessing 30 recurrent fusion drivers using RNA-based analysis. The panel includes clinically relevant genes involved in MPNs, myelodysplastic neoplasms, acute myeloid leukemia, and related myeloid disorders (Supplementary Table S2). For the purposes of this study, cases were classified as “driver-positive” when a canonical MPN driver alteration (*BCR::ABL1* fusion or a pathogenic *JAK2*, *CALR*, or *MPL* alteration) was identified by the validated Oncomine Myeloid Assay and as “driver-negative” when none of these canonical driver alterations were detected. This classification was used solely for the purposes of this study and does not necessarily correspond to routine clinical laboratory reporting terminology. Sequencing data were processed using the Genexus Software Suite version 6.6 (Thermo Fisher Scientific, Waltham, MA, USA) with automated alignment, variant calling, quality assessment, and annotation.

For cases with additional bone marrow analysis, clinical comprehensive molecular profiling was performed using Archer/IDT Variantplex Myeloid panel (Archer/IDT, Boulder, CO, USA), a DNA-only 72 gene panel, and sequenced using the Illumina NextSeq sequencer (Illumina, San Diego, CA, USA). Data analysis was performed using the Archer Analysis platform version 6.2 with secondary analysis using an in-house bioinformatics platform.

Sequence variants were interpreted according to established AMP/ASCO/CAP recommendations using publicly available databases. Only pathogenic and likely pathogenic variants were included in the present analysis, whereas variants of uncertain significance were excluded unless otherwise specified. Variants were reported according to the laboratory’s validated assay performance characteristics, including the validated variant allele frequency (VAF) detection threshold of above 1% for *JAK2*, *CALR*, *MPL*, and *KIT*. Molecular findings were not interpreted in isolation but were integrated with the patient’s clinical presentation, laboratory findings, morphologic evaluation, cytogenetic studies, and follow-up data to assess their diagnostic relevance and to distinguish potential age-related clonal hematopoiesis (CHIP) from clinically significant myeloid neoplasms.

### 2.3. Statistical Analysis

Statistical analyses were conducted using Rstudio version 2024.04.2+764 (Posit Software, PBC, Boston, MA, USA) with R version 4.4.1 (R Foundation for Statistical Computing, Vienna, Austria) and Microsoft Excel. Fisher’s Exact Test was employed to compare the differences in frequencies between groups, while categorical variables were compared using the chi-square test. Fisher’s test was performed using “fisher.test” function in “stats” package in R, whereas chi-squared test was done using “chisq.test” function from the same package. Visualizations, including bar diagrams, pie charts, and variant distribution heatmaps, were created using the “ggplot2” package in R (R version 4.4.1; R Foundation for Statistical Computing, Vienna, Austria). Additional heatmaps for gene mutations and disorders were generated using the “heatmap” function in the “stats” package in R. Correlation plot of variant allele frequencies were produced in Microsoft Excel. The scripts used for the analysis are available publicly on Github: https://github.com/manoj34sapkota/Tirzo_et_al/ (last accessed on 15 June 2026).

## 3. Results

### 3.1. Patient Characteristics

Peripheral blood samples from 1209 patients (*n* = 618 male, *n* = 591 female; mean age at presentation = 58 years; range 1 to 99 years) were tested. The indications for testing included cytosis, clinical concern for or history of myeloproliferative neoplasm (MPN), cytopenia, and a combination of cytopenia and cytosis. Clinical concern for MPN encompassed patients with thrombotic events (such as deep vein thrombosis, pulmonary embolism, or cerebral venous thrombosis), splenomegaly, or other unspecified clinical findings (Table 1).

### 3.2. Distribution of Canonical MPN Driver Alterations

The results were categorized based on the presence or absence of canonical MPN driver alterations, including *BCR::ABL1* fusions, pathogenic *JAK2* mutations (including V617F and exon 12 mutations), pathogenic *CALR* mutations, and pathogenic *MPL* mutations identified by the validated clinical assay. A total of 141 patients tested positive for at least one of the main drivers: 103 patients with *JAK2*, 18 with *CALR*, 16 with *BCR::ABL1* and 13 with *MPL*. Nine patients had more than one mutation in driver genes. Samples from 1068 patients were reported driver-negative after testing (Table 1).

The median age of the driver-negative group was slightly lower compared to the driver-positive group, 58 and 69 years respectively, with no statistically significant difference in sex between the two groups.

### 3.3. Additional Pathogenic Mutations Identified by Comprehensive NGS

**Figure 1:**
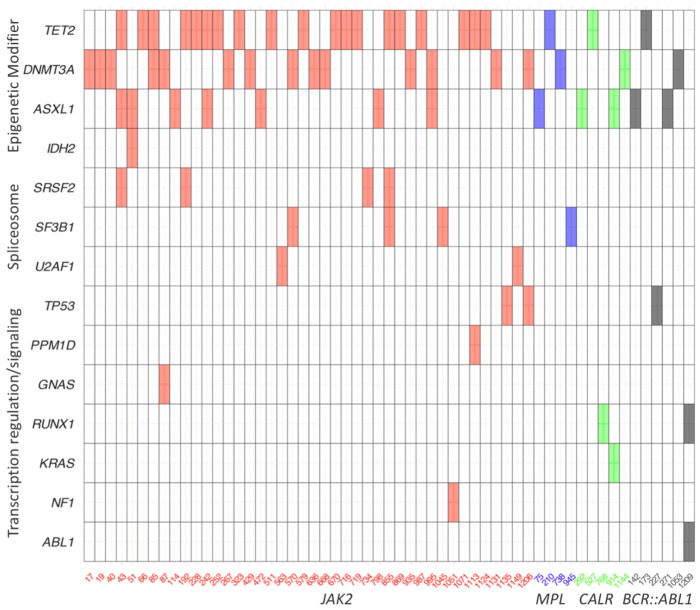
The heatmap illustrates variant distribution for all patients with paired samples according to MPN driver mutation. Each column represents an individual patient and is grouped according to the corresponding driver mutation. Colored cells indicate the presence of a pathogenic or likely pathogenic mutation in the corresponding gene (*JAK2*: red, *MPL*: blue, *CALR*: green, *BCR::ABL1*: gray); colors denote the canonical driver mutation group.

Retrospective analysis showed 251 patients (21%) harbored at least one unreported pathogenic mutation in at least one gene. Importantly, many of these clinically relevant mutations were identified at the time of initial molecular evaluation, potentially shortening the interval to comprehensive genomic characterization. A 141 cases were driver-positive for one of the MPN genes, and 57 of 141 (40%) had mutations identified in myeloid malignancies beyond the primary drivers, including the most frequently identified clinically relevant pathogenic co-mutations, involving epigenetic regulators (*TET2*, *DNMT3A*, *ASXL1*, *IDH2*), splicing factors (*SRSF2*, *SF3B1*, *U2AF1*), transcription factors and signaling pathway genes (*TP53*, *RUNX1*, *PPM1D*, *NF1*, *NRAS*, *KRAS*, *GNAS*) (Figure 1).

### 3.4. High-Molecular-Risk Mutations

Out of the 141 driver-positive cases, 23 contained genes classified as high molecular risk or indicative of patients at risk for fibrotic progression or leukemic transformation within the spectrum of MPNs [18,21,22]. These included 12 *ASXL1*, 3 *TP53*, 4 *SRSF2*, 4 *SF3B1*, 2 patients with *RUNX1*, and 2 with *U2AF*1 (one patient with Q157 mutation), as well as 1 case with *IDH2*. Three patients had two or more of these mutations concurrently at the time of testing.

### 3.5. Additional Pathogenic Mutations in Canonical MPN Driver-Negative Cases

**Figure 2:**
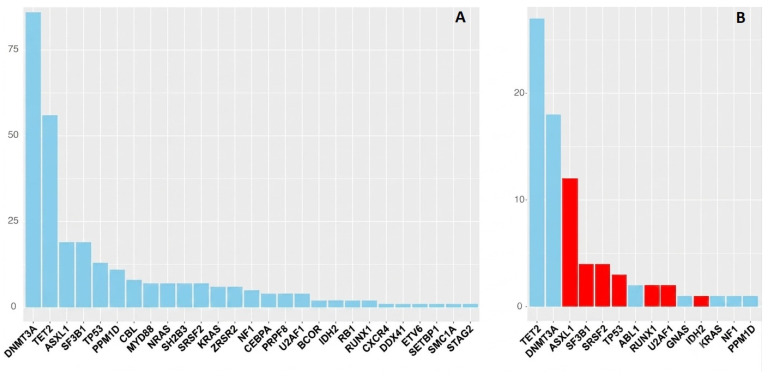
Frequency and distribution of additional mutations in reported driver-negative (**A**) and driver-positive cases with high-risk mutation genes in red (**B**).

Out of 1068 reported driver-negative cases, 194 (18%) had at least one unreported pathogenic mutation, and the distribution of the affected genes showed a broader range, with several genes commonly mutated across different myeloid malignancies (Figure 2).

### 3.6. Clinical Correlations

The clinical indication for testing was significantly associated with the presence of additional pathogenic mutations in both the driver-positive and driver-negative groups. Specifically, *DNMT3A* and *TET2* mutations were significantly enriched among patients evaluated for cytosis in the driver-positive cohort (*p* < 0.001).

Patients harboring previously unreported pathogenic mutations were more likely to undergo concurrent or subsequent bone marrow biopsy and to receive a diagnosis of a hematologic malignancy (13%; 33/251) compared with patients without additional pathogenic mutations (6.5%; 63/958) (Supplementary Table S1). After excluding the 15 driver-positive cases without additional pathogenic mutations, this rate decreased to 5%. Because this was a retrospective observational study, these findings represent an association and should not be interpreted as evidence that independently influenced clinical decision-making.

### 3.7. Peripheral Blood–Bone Marrow Concordance

**Figure 3:**
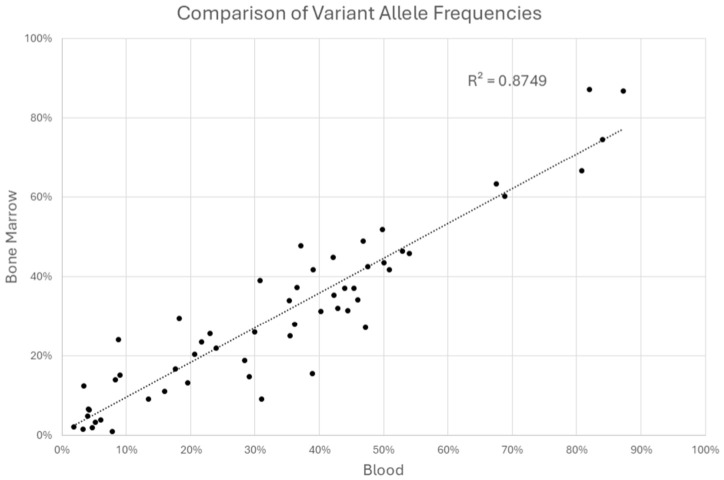
Correlation of mutation variant allele frequency (VAF): Bone Marrow and Peripheral blood. Of note, different panels and sequencing platforms were used for BM verses PB (see Section 2), which will skew the VAFs. The dashed line indicates the linear regression (best-fit) line.

To determine whether peripheral blood (PB) serves as a reliable matrix for the early detection and screening of genetic mutations associated with hematological diseases, we compared genetic data between bone marrow (BM) and PB samples. Our findings indicate a high degree of concordance in mutation detection between the two sample types, with a correlation coefficient (R^2^) of 0.87 (Figure 3).

### 3.8. SH2B3 Variants

During analysis of the sequencing data, *SH2B3* variants were identified in 85 patients. Of these, 11 cases harbored pathogenic or likely pathogenic (P/LP) variants and/or variants of uncertain significance (VUS), classified according to AMP/ACMG guidelines. Notably, several *SH2B3* variants exhibited allele frequencies near 50%, raising the possibility of germline origin (Supplementary Table S2).

## 4. Discussion

Mutational status has become increasingly crucial in clinical practice. Initially, research on gene mutations in MPNs focused particularly on the *BCR::ABL1*, *JAK2*, *CALR*, and *MPL* genes and their roles in disease development [23]. However, with advancements in molecular testing and access to broader panels, it is now evident that the MPN phenotype is not determined solely by the primary driver mutations. Numerous additional mutations beyond these primary drivers have been identified in MPNs and are categorized into several gene families including epigenetic regulators (*ASXL1*, *EZH2*, *TET2*, *IDH1/2*, *DNMT3A*), spliceosome components (*SRSF2*, *SF3B1*, *U2AF1*, *ZRSR2*), transcriptional regulators (*TP53*, *RUNX1*, *IKZF1*), general cell signaling genes (*KRAS*, *PTPN11*), and specific negative regulators of JAK/STAT signaling (*CBL*) [24,25]. Additionally, factors such as allele burden and the order in which driver and other mutations are acquired have been identified as significant contributors to phenotypic differences [15,26,27].

In our study, the distribution of additional concurrent mutations observed at diagnosis aligned with the expected patterns reported by previous studies. Our findings are consistent with previous targeted sequencing studies demonstrating that the most frequent co-occurring mutations in MPNs involve genes regulating epigenetic modification (*TET2*, *DNMT3A*, and *ASXL1*), RNA splicing (*SRSF2*, *SF3B1*, and *U2AF1*), and transcriptional regulation or signaling (*TP53*, *RUNX1*, and *NRAS*). Similarly to these reports, additional mutations in our cohort were observed across different canonical driver groups and frequently involved genes with established prognostic significance. Furthermore, the identification of high-molecular-risk mutations in a substantial proportion of driver-positive patients supports previous observations that comprehensive genomic profiling provides clinically relevant information beyond the canonical driver mutations alone [11,18,19]. Although these mutations are not exclusive to MPN and are found across various myeloid malignancies [28].

A clustering analysis showed that most mutations in driver-positive cases involved genes with known prognostic significance, supporting the panel’s utility as a screening tool. Specifically, *DNMT3A* and *TET2* mutations were significantly associated with cytosis (*p* < 0.001), suggesting a potential role in modifying the myeloproliferative phenotype and highlighting the relevance of non-driver mutations in disease presentation and risk stratification. Importantly, these mutations were identified at the time of the initial molecular evaluation, before definitive morphologic classification, allowing comprehensive genomic characterization early in the diagnostic workup. This early identification may help prioritize patients for further hematologic evaluation, including bone marrow examination, while simultaneously providing additional relevant molecular information.

While certain mutations appeared to cluster within the driver-positive reported group, the overall mutational landscape was heterogeneous and not limited to a single driver gene, among the driver-negative reported group.

Historically, prognostic scoring systems such as International Prognostic Scoring System (IPSS), Dynamic International Prognostic Scoring System (DIPSS), and DIPSS Plus have mainly relied on clinical parameters without incorporating driver mutations as prognostic factors [29,30,31]. These models have often struggled to effectively distinguish between intermediate-2 and high-risk patients, particularly in pre-primary myelofibrosis (pre-PMF) and overt myelofibrosis (MF), which differentiation is crucial for making informed decisions regarding high-risk interventions, such as stem cell transplantation. The discovery of new mutations and their implications for patient outcomes have led to the development of mutation-enhanced risk scores. The most recent Mutation-Enhanced International Prognostic Scoring System 70 (MIPSS70)-Plus version 2.0 has significantly improved predictive accuracy in PMF patients. Notably, this system includes high-molecular-risk (HMR) mutations, such as *ASXL1*, *EZH2*, *SRSF2*, *IDH1/*2, and *U2AF1*, which are associated with statistically significant higher cumulative incidences of adverse outcomes compared to the low-molecular-risk (LMR) category (no mutations in the HMR genes). Additionally, the system considers the number of mutations in the scoring to enhance prognostic accuracy [18,32]. Similarly, spliceosome mutations like *SRSF2*, *SF3B1*, and *U2AF1* in ET, along with *SRSF2* in PV, have been linked to clinical outcomes. Moreover, *TP53* mutations are critically implicated in leukemic transformation in ET. These findings have led to the integration of these mutations into prognostic scoring systems [22]. Our research identified these unreported high-risk mutations in 40% of cases with positive additional mutations, with more than 2 HMR mutations noted in 3 cases. This presence led to challenges such as prognostic uncertainty, suboptimal treatment strategies, delayed recognition of disease progression, and potential misdiagnosis in some instances.

Multiple studies have demonstrated high concordance between peripheral blood and bone marrow NGS findings in myeloid neoplasms, supporting peripheral blood as a reliable screening source [33,34]. Our study demonstrated a high level of agreement in mutation detection between peripheral blood (PB) and bone marrow (BM). The targeted sequencing of PB showed a high negative predictive value (NPV) of 100%, effectively identifying individuals at very low risk of a myeloid neoplasm and potentially sparing them from invasive and costly BM assessments. The positive predictive value (PPV) was 90.9%. Overall, indicating that PB testing can reliably predict the presence of a myeloid neoplasm in BM. However, although recurrent single-gene mutations were identified in some cases, the frequency of some individual gene mutations was too low to provide convincing evidence of their association with the disease. These findings support the use of peripheral blood NGS as a practical first-line molecular screening strategy in appropriately selected patients, potentially reducing unnecessary invasive bone marrow procedures during the initial diagnostic evaluation. However, because CHIP becomes increasingly common with advancing age, isolated pathogenic variants identified by peripheral blood NGS should not be interpreted as diagnostic of an underlying myeloid neoplasm. Rather, molecular findings must always be integrated with the patient’s clinical presentation, hematologic parameters, morphologic evaluation, cytogenetic findings, and follow-up data to distinguish CHIP from clinically significant myeloid disease. Accordingly, peripheral blood molecular testing should complement rather than replace bone marrow examination, as morphologic assessment remains indispensable for definitive disease classification, particularly in distinguishing ET from pre-fibrotic primary myelofibrosis and for assessing marrow fibrosis and blast burden.

Unmasking additional mutations can not only aid in prognostic stratification but also address the so-called “Triple-negative” MPNs, which lack mutations in all four main driver genes. Non-driver mutations can offer potential evidence of clonality, thus providing significant diagnostic and prognostic value. In addition to improving prognostic scoring systems extended genetic testing can also help predict how patients will respond to targeted therapies such as ruxolitinib, the only approved medication for myelofibrosis [12,13].

In addition to somatic mutations, the detection of known germline variants like *SH2B3* in younger individuals within our cohort points to a potential hereditary predisposition to MPNs in some cases. However, our current testing approach does not distinguish between germline and somatic mutations. Therefore, further germline-specific testing may be advisable.

This study has several limitations. As a retrospective single-institution analysis of a real-world clinical cohort that included both patients undergoing initial evaluation and those with previously established MPNs, the findings require validation in larger prospective multicenter studies. Furthermore, because this study reflects routine clinical practice, not all patients underwent bone marrow examination or had complete longitudinal follow-up, and the additional molecular findings were identified only through retrospective analysis rather than being available to treating physicians. Consequently, the observed association with subsequent bone marrow evaluation should not be interpreted as causal. Finally, although the targeted panel included the major clinically relevant myeloid genes, alterations outside the assay design were not evaluated.

## 5. Conclusions

Our institutional cohort suggests that peripheral blood NGS provides a reliable complementary molecular screening strategy for identifying patients likely to harbor an underlying myeloid neoplasm while simultaneously generating diagnostically and prognostically relevant genomic information beyond conventional stepwise PCR testing. Although targeted testing for canonical driver mutations remains appropriate in many patients with typical clinical presentations, comprehensive NGS panels offer particular value in diagnostically challenging cases, including triple-negative MPNs, by establishing clonality, identifying additional high-molecular-risk mutations, and quantifying allelic burden. These molecular data improve diagnostic precision, refine risk stratification, and increasingly contribute to individualized patient management. Furthermore, our findings suggest that peripheral blood NGS may serve not only as a reliable diagnostic tool but also as a practical platform for longitudinal molecular monitoring. Nevertheless, molecular findings should always be interpreted in conjunction with clinical, laboratory, and morphologic features, as bone marrow examination remains the gold standard for definitive MPN classification. In addition to their established prognostic significance, high-risk molecular alterations are being incorporated into therapeutic decision-making by identifying patients who may benefit from earlier allogeneic hematopoietic stem cell transplantation, enrollment in mutation-informed clinical trials, or emerging targeted therapies [32,35]. Prospective studies are needed to confirm the prognostic significance of these mutations identified at diagnosis in patients without at-risk clinical conditions, such as myelofibrosis.

## Figures and Tables

**Table 1:** Case indications and results from the MPN panel. Canonical driver alteration detected refers to the presence of a *BCR::ABL1* fusion or a pathogenic *JAK2*, *CALR*, or *MPL* mutation. No canonical driver alteration detected indicates that none of the aforementioned driver alterations were identified by the clinical assay. This classification was used for the purposes of this study and does not necessarily correspond to routine clinical laboratory reporting terminology.

Total	1209	
Indication	Concern	442
	Cytopenia	92
	Cytosis	634
	Cytosis/cytopenia	41
Canonical driver alteration detected	141	
	*BCR::ABL1*	16
	*JAK2*	103
	*MPL*	13
	*CALR*	18
	2+ variants	9
No canonical driver alteration detected	1068	
	Mutation present	194
	Mutation absent	874

## Data Availability

For original data, please contact celeste.eno@csmc.edu.

## Supplementary Materials

The following supporting information can be downloaded at: https://www.mdpi.com/article/10.3390/cancers18152458/s1, Table S1: Individual patient-level clinical and molecular data for the study; Table S2: *SH2B3* variants identified in the study cohort.
