# Supplementary material for: Unmasking Additional Mutations: A Single Institution Study in Myeloproliferative Neoplasms
Source: Cancers (Basel). 2026 Jul 31;18(15):2458. doi: 10.3390/cancers18152458 (PMC13465104; doi:10.3390/cancers18152458)
Supplement: Supplementary file 1 [file cancers-18-02458-s001.zip › cancers-4416726-supplementary.pdf]

Supplementary Data

Table S1. Individual patient-level clinical and molecular data for the study cohort undergoing peripheral blood myeloid next-generation sequencing. The table includes age, sex, indication for testing, identified driver mutation(s), additional pathogenic or likely pathogenic mutations, variant allele frequency (VAF) of additional mutations, and the final clinical diagnosis assigned after comprehensive clinicopathologic evaluation. Blank fields indicate that no additional clinically significant mutation or corresponding clinical information was available. Abbreviations: VAF, variant allele frequency; MPN, myeloproliferative neoplasm; ET, essential thrombocythemia.

Pt ID	Age	Sex	Indication for testing	Driver	Additional mutations	VAF	Final Diagnosis
1	70	F	cytopenia				NA
2	85	F	cytosis		*SF3B1*	<5%	Lymphoma
3	40	F	cytosis				NA
4	79	F	cytosis	*JAK2*			MPN (ET)
5	68	M	cytosis				NA
6	73	M	cytosis		*TET2*	16.50 %	MDS (clinical)
6					*NF1*	8.50%	
6					*NRAS*	<5%	
7	78	M	cytopenia				NA
8	34	M	cytosis				NA
9	49	F	cytosis				NA
10	38	M	cytosis				NA
11	86	F	cytosis				NA
12	21	M	concern				NA
13	39	F	cytosis				NA
14	54	F	cytosis		*MYD88*	5.20%	Plasma cell neoplasm
15	86	M	cytosis				NA
16	64	M	cytosis		*TET2*	<5%	Clonal hematopoiesis
17	75	F	cytosis	*JAK2*	*DNMT3A*	6.20%	MPN (ET)
18	72	M	cytosis		*PPM1D*	22.40 %	MDS (clinical)
19	75	M	cytosis	*JAK2*	*DNMT3A*	21.90 %	MPN (ET)
20	34	F	cytosis				NA
21	51	M	cytosis	*JAK2*			MPN (PV)
22	99	M	cytosis		*DNMT3A*	<5%	Clonal hematopoiesis
23	92	F	cytopenia				NA
24	81	M	cytosis		*SF3B1*	<5%	Clonal hematopoiesis
25	36	F	cytosis				NA
26	51	F	cytosis				NA
27	30	F	cytosis				NA

28	70	M	cytosis	*JAK2*			MPN (PV)
29	26	M	cytosis				NA
30	75	F	cytopenia		*TP53*	86.90 %	MPN/MDS
30							
31	61	M	concern		*PRPF8*	5.80%	Clonal hematopoiesis
32	62	M	cytosis				NA
33	1	F	cytosis				Histiocytic neoplasm
34	51	M	cytosis				NA
35	99	F	cytosis/cytopenia				NA
36	41	M	cytosis				NA
37	37	F	cytosis				NA
38	81	M	cytosis	*JAK2*			MPN (ET)
39	70	M	cytosis				NA
40	70	M	MPN	*JAK2*	*DNMT3A*	12.70 %	MPN (ET)
41	71	M	cytosis				NA
42	48	F	cytosis				NA
43	91	M	cytosis	*JAK2*	*TET2*	78.00 %	MPN/MDS
43					*ASXL1*	14.80 %	
43					*SRSF2*	53.10 %	
44	39	M	cytosis				NA
45	80	M	cytopenia				NA
46	78	F	cytopenia				NA
47	58	M	cytosis				NA
48	73	F	concern		*DNMT3A*	<5%	Clonal hematopoiesis
49	65	F	MPN				NA
50	82	F	cytosis	*JAK2*			MPN (PV)
51	77	M	cytosis/cytopenia	*JAK2*	*IDH2*	42.80 %	MPN (NOS)
51					*ASXL1*	38.90 %	
52	78	M	cytosis				NA
53	41	M	cytosis	*BCR::ABL1*			MPN (CML)
54	70	M	cytosis				NA
55	83	F	cytosis				NA
56	62	M	cytopenia				Plasma cell neoplasm
57	68	M	cytosis/cytopenia				NA
58	50	M	cytopenia				NA
59	35	F	cytosis				NA
60	73	M	cytopenia				NA
61	61	M	cytosis	*JAK2*			MPN (ET)
62	63	M	cytosis				NA

63	59	F	cytopenia				NA
64	53	M	cytosis/cytopenia				NA
65	53	F	cytosis/cytopenia				NA
66	77	F	cytosis	*JAK2*	*TET2*	41.10%	MPN (ET)
67	95	F	cytosis		*DNMT3A*	<5%	NA
68	25	M	cytosis				NA
69	26	F	cytosis				NA
70	42	F	cytosis/cytopenia				NA
71	57	M	cytosis				NA
72	53	F	concern				Plasma cell neoplasm
73	74	F	cytosis				Lymphoma
74	66	F	concern				NA
75	48	M	MPN	*MPL*	*ASXL1*	<5%	MPN (MF)
75				*JAK2*			
76	24	M	concern				NA
77	86	F	cytosis/cytopenia		*TET2*	50.30%	MPN/MDS
77					*ASXL1*	16.00%	
77					*NRAS*	27.20%	
77					*NRAS*	14.10%	
78	49	F	cytopenia				NA
79	55	M	cytopenia		*DNMT3A*	31.6%	MDS (clinical)
79					*DNMT3A*	<5%	
80	43	M	concern				NA
81	36	M	cytopenia				NA
82	61	F	cytosis		*DDX41*	69.50%	Germline origin
83	68	M	concern				NA
84	64	M	cytosis/cytopenia				NA
85	93	F	cytosis	*JAK2*	*DNMT3A*	42.70%	MPN (NOS)
85					*TET2*	<5%	
86	38	M	cytopenia				NA
87	72	F	MPN	*JAK2*	*DNMT3A*	5.40%	MPN (ET)
87					*GNAS*	5.30%	
88	36	M	cytosis				NA
89	61	M	cytosis				NA
90	86	F	cytosis		*PRPF8*	<5%	Clonal hematopoiesis
90					*DNMT3A*	<5%	
91	50	M	cytosis				NA
92	44	F	cytosis		*SH2B3*	<5%	Clonal hematopoiesis

93	74	M	cytosis				NA
94	34	M	cytosis				NA
95	25	F	cytosis				NA
96	79	M	cytosis		*PPM1D*	5.60%	Clonal hematopoiesis
96					*DNMT3A*	<5%	
96					*DNMT3A*	17.10 %	
97	40	M	cytosis				NA
98	76	M	cytosis/cytopeni a		*ZRSR2*	<5%	Clonal hematopoiesis
99	35	M	concern				NA
100	57	F	cytosis				NA
101	40	F	concern				NA
102	77	M	concern				NA
103	81	M	cytosis				NA
104	67	M	cytosis		*DNMT3A*	<5%	Clonal hematopoiesis
105	29	F	cytosis				NA
106	48	F	cytosis				NA
107	62	M	cytosis				NA
108	52	F	cytosis		*NRAS*	<5%	MPN (NOS)
109	39	M	cytosis				NA
110	45	F	concern				NA
111	61	F	cytosis		*DNMT3A*	<5%	Clonal hematopoiesis
112	80	M	cytosis	*JAK2*			MPN (NOS)
113	92	M	concern		*ZRSR2*	6.70%	Clonal hematopoiesis
113					*ZRSR2*	7.50%	
114	81	M	cytosis	*JAK2*	*ASXL1*	21.60 %	MPN (ET)
115	80	M	MPN	*JAK2*			MPN (ET)
116	47	M	cytosis				NA
117	45	M	cytosis				NA
118	59	F	cytosis				NA
119	27	F	concern				NA
120	67	M	cytosis				NA
121	54	M	cytosis				NA
122	30	F	concern				NA
123	49	F	cytosis				NA
124	42	F	cytosis				NA
125	39	F	cytosis				NA
126	35	M	concern				NA
127	58	F	concern				NA
128	63	F	concern				NA
129	75	M	cytosis				NA
130	64	F	cytosis		*PPM1D*	15.10 %	Clonal hematopoiesis

131	95	F	cytosis	*MPL*			MPN (MF)
132	43	F	cytosis				NA
133	73	M	cytosis				NA
134	72	F	cytosis	*JAK2*			MPN (PV)
135	58	F	cytopenia				NA
136	26	M	cytosis				NA
137	52	F	cytosis				NA
138	28	F	cytosis	*JAK2*			MPN (ET)
139	45	M	cytosis				NA
140	61	M	cytosis				NA
141	38	F	cytosis				NA
142	65	M	cytosis	*BCR::ABL1*	*ASXL1*	43.30 %	MPN (CML)
143	32	F	cytosis				NA
144	47	F	cytosis				NA
145	35	F	concern				NA
146	29	F	cytosis				NA
147	76	F	cytosis		*DNMT3A*	<5%	Clonal hematopoiesis
148	39	M	cytosis				NA
149	55	M	concern				NA
150	76	M	cytosis				NA
151	67	M	cytosis	*JAK2*			MPN (NOS)
152	48	F	cytosis				NA
153	27	M	cytosis				NA
154	73	F	concern		*RB1*	53.20 %	Hereditary hematologic predisposition
155	46	F	concern				NA
156	76	M	cytosis		*ZRSR2*	60.40 %	MPN/MDS
156					*CBL*	16.80 %	
156					*CBL*	11.20 %	
156					*IDH2*	31.30 %	
156					*SRSF2*	34.10 %	
156					*TET2*	15.30 %	
156					*TET2*	14.00 %	
156					*ZRSR2*	29.70 %	
157	34	F	concern				NA
158	21	F	MPN	*JAK2*			MPN (PV)
159	68	F	MPN				NA
160	87	F	concern		*DNMT3A*	9.80%	Clonal hematopoiesis
161	75	M	cytopenia				NA

162	24	M	cytosis				NA
163	77	F	MPN	*CALR*			MPN (ET)
164	65	M	concern				NA
165	81	F	concern				NA
166	44	F	cytosis				NA
167	69	M	MPN	*JAK2*			MPN (ET)
168	77	F	cytosis		*SF3B1*	45.00 %	MPN/MDS
168					*ASXL1*	14.90 %	
168					*NRAS*	35.40 %	
169	40	F	cytosis		*CEBPA*	<5%	MDS (clinical)
169					*DNMT3A*	93.40 %	
170	59	F	cytosis		*BCOR*	<5%	Clonal hematopoiesis
171	64	F	cytosis				NA
172	66	M	cytosis		*DNMT3A*	<5%	Clonal hematopoiesis
173	55	M	concern	*BCR::ABL1*	*TET2*	<5%	MPN (CML)
174	87	M	cytosis				NA
175	83	M	cytosis		*PPM1D*	<5%	Clonal hematopoiesis
175					*SF3B1*	<5%	
176	71	M	cytopenia		*TET2*	<5%	Clonal hematopoiesis
176					*PPM1D*	14.40 %	
177	81	F	concern		*DNMT3A*	<5%	Clonal hematopoiesis
178	34	F	cytosis				NA
179	87	M	cytopenia				NA
180	52	F	cytosis				NA
181	67	F	concern				NA
182	29	F	cytosis				NA
183	74	F	cytosis	*JAK2*			MPN (NOS)
184	56	M	cytosis				NA
185	85	M	cytosis				NA
186	65	M	cytosis				NA
187	74	M	cytosis				NA
188	67	M	cytosis		*DNMT3A*	<5%	Clonal hematopoiesis
188					*DNMT3A*	<5%	
189	79	F	cytopenia				NA
190	80	M	cytopenia		*ASXL1*	<5%	Clonal hematopoiesis
190					*MYD88*	1.30%	
191	44	F	cytosis				NA
192	67	F	MPN	*JAK2*	*TET2*	24.30 %	MPN (ET)
192					*SRSF2*	5.20%	
193	28	M	cytosis				NA

194	51	F	cytosis/cytopenia				NA
195	72	M	cytosis				NA
196	75	M	cytosis		*DNMT3A*	<5%	Clonal hematopoiesis
197	78	M	cytosis				NA
198	52	M	cytosis				NA
199	60	M	cytopenia				NA
200	66	M	cytosis		*SRSF2*	26.10%	Lymphoma
200					*CXCR4*	25.10%	
200							
201	32	F	cytosis				NA
202	44	M	concern				NA
203	82	M	concern		*PPM1D*	<5%	Clonal hematopoiesis
203					*SF3B1*	<5%	
204	70	M	MPN				NA
205	63	F	MPN	*JAK2*			MPN (PV)
206	75	F	cytosis/cytopenia				NA
207	75	M	cytosis				NA
208	87	F	cytopenia				NA
209	54	F	cytosis				NA
210	77	F	cytosis	*MPL*	*TET2*	<5%	MPN (ET)
211	65	F	cytosis				NA
212	78	M	cytosis				NA
213	37	F	cytosis	*JAK2*			MPN (PV)
214	82	F	cytosis				NA
215	72	M	cytopenia				NA
216	72	M	cytosis				NA
217	85	F	cytosis				NA
218	48	M	cytopenia				NA
219	83	M	cytosis/cytopenia		*DNMT3A*	27.00%	MDS (clinical)
219					*TET2*	18.40%	
219					*TET2*	6.80%	
220	82	M	cytosis		*DNMT3A*	<5%	Clonal hematopoiesis
221	61	F	cytosis	*JAK2*			MPN (ET)
222	86	M	cytosis		*DNMT3A*	<5%	Clonal hematopoiesis
223	58	M	cytosis				NA
224	64	M	cytosis/cytopenia		*DNMT3A*	11.40%	MDS (clinical)
225	85	F	concern		*ASXL1*	<5%	Clonal hematopoiesis
226	72	M	concern		*DNMT3A*	<5%	Clonal hematopoiesis
227	66	M	cytosis	*BCR::ABL1*	*TP53*	45.90%	MPN (CML)

227							
228	80	F	cytosis	*JAK2*	*TET2*	<5%	MPN (NOS)
229	70	F	cytosis				NA
230	60	F	cytosis	*JAK2*			MPN (NOS)
231	74	M	cytosis				NA
232	83	F	cytosis		*DNMT3A*	<5%	Clonal hematopoiesis
233	34	M	cytosis				NA
234	75	M	cytosis				NA
235	33	F	cytosis				NA
236	42	F	concern				NA
237	48	M	cytosis				NA
238	54	F	concern				NA
239	53	M	concern				NA
240	63	M	cytopenia				NA
241	61	F	cytosis				NA
242	92	F	cytosis	*JAK2*	*ASXL1*	28.40 %	MPN (NOS)
242					*TET2*	<5%	
243	35	F	cytosis				NA
244	88	F	cytopenia		*TET2*	19.00 %	MDS (clinical)
245	91	F	cytopenia		*DNMT3A*	<5%	Clonal hematopoiesis
246	69	M	concern				NA
247	29	F	cytosis				NA
248	84	F	concern		*TET2*	6.80%	Clonal hematopoiesis
249	86	F	concern		*TP53*	<5%	Clonal hematopoiesis
250	59	M	cytosis				Lymphoma
251	72	F	cytosis				NA
252	92	F	MPN	*JAK2*	*TET2*	53.50 %	MPN (PV)
253	53	F	cytopenia				NA
254	75	M	cytosis				NA
255	88	F	concern		*ASXL1*	34.50 %	MDS (clinical)
256	46	M	cytosis				NA
257	52	F	cytosis				NA
258	86	M	cytosis		*MYD88*	29.00 %	Lymphoma
259	41	F	cytopenia				NA
260	45	M	concern				NA
261	87	M	concern		*TET2*	<5%	Clonal hematopoiesis
262	35	M	concern				NA
263	48	M	MPN	*MPL*			MPN (MF)
263				*JAK2*			
264	32	F	cytosis				NA
265	47	M	concern				NA

266	41	M	cytosis				NA
267	82	M	cytosis	*JAK2*	*DNMT3A*	<5%	Clonal hematopoiesis
268	42	F	cytosis				NA
269	78	F	cytosis				NA
270	89	F	cytosis		*ASXL1*	<5%	Clonal hematopoiesis
270					*TET2*	<5%	
271	68	F	cytosis	*BCR::ABL1*	*ASXL1*	27.60 %	MPN (CML)
272	76	M	concern		*ASXL1*	12.20 %	Plasma cell neoplasm
273	37	F	cytosis				NA
274	53	F	cytosis				NA
275	89	M	cytopenia		*SRSF2*	31.60 %	MDS
275					*ASXL1*	<5%	
275					*RUNX1*	12.50 %	
275					*SETBP1*	<5%	
275							
276	61	M	cytopenia				NA
277	83	M	cytopenia		*TET2*	44.40 %	NA
278	75	M	cytosis				NA
279	29	F	concern				NA
280	53	M	concern				NA
281	36	M	cytosis				NA
282	47	F	concern				NA
283	78	F	cytosis				NA
284	42	M	concern				NA
285	76	M	cytosis				NA
286	63	M	cytosis		*TET2*	<5%	Clonal hematopoiesis
287	59	M	concern				NA
288	56	F	cytosis				NA
289	53	M	concern				NA
290	74	M	concern				NA
291	33	F	concern				NA
292	59	M	MPN	*CALR*	*ASXL1*	39.00 %	MPN (PMF)
293	68	M	cytosis/cytopeni a		*TP53*	<5%	Clonal hematopoiesis
294	69	F	concern		*DNMT3A*	39.80 %	MDS (clinical)
295	77	F	cytosis				NA
296	47	F	concern				NA
297	85	F	cytosis/cytopeni a		*SF3B1*	29.50 %	MDS
298	80	F	cytosis/cytopeni a				NA

299	75	F	cytosis				NA
300	44	M	cytosis				NA
301	70	F	cytosis		*TP53*	<5%	Clonal hematopoiesis
302	82	F	cytosis				NA
303	55	M	cytosis				NA
304	32	F	cytosis				NA
305	63	M	cytosis				NA
306	33	F	concern				NA
307	87	M	cytopenia		*NF1*	29.00 %	Hereditary hematologic predisposition
308	51	M	concern				NA
309	54	M	cytosis				NA
310	71	F	cytopenia				NA
311	60	F	cytosis		*PPM1D*	<5%	Clonal hematopoiesis
312	24	F	cytosis				NA
313	76	M	cytosis				NA
314	35	M	cytosis				NA
315	70	M	cytopenia				NA
316	27	M	concern				NA
317	24	F	concern				NA
318	20	M	concern				NA
319	33	M	cytosis				NA
320	63	M	cytopenia		*TET2*	34.70 %	MDS (clinical)
321	61	F	cytosis				NA
322	68	M	cytosis				NA
323	68	F	cytosis	*JAK2*	*TET2*	44.20 %	MPN (PV)
324	64	M	cytosis				NA
325	69	F	concern				NA
326	60	M	cytosis	*JAK2*			MPN (ET)
327	75	M	concern				NA
328	58	F	concern				NA
329	49	F	cytosis				NA
330	85	F	cytosis	*JAK2*			MPN (PV)
331	73	M	concern		*NF1*	<5%	Clonal hematopoiesis
331					*DNMT3A*	<5%	
332	49	F	MPN	*JAK2*			MPN (ET)
333	76	M	concern				NA
334	48	M	cytosis				NA
335	76	M	cytosis		*NRAS*	44.40 %	MPN/MDS
336	79	M	concern		*TET2*	<5%	Clonal hematopoiesis
337	25	M	cytosis				NA
338	68	M	cytosis	*JAK2*			MPN (NOS)

339	71	M	cytosis				NA
340	71	M	concern				NA
341	38	F	concern				NA
342	68	M	concern		*SF3B1*	<5%	Clonal hematopoiesis
342					*NF1*	<5%	
343	45	M	cytosis				NA
344	58	M	cytosis		*CBL*	<5%	Clonal hematopoiesis
345	73	F	concern				NA
346	58	M	concern				NA
347	52	M	cytosis				NA
348	65	M	cytosis	*JAK2*			MPN (MF)
348							
349	55	F	cytosis				NA
350	52	F	cytosis				NA
351	39	M	cytopenia				NA
352	67	F	cytosis				NA
353	76	M	concern		*SH2B3*	<5%	Clonal hematopoiesis
353					*TET2*	9.70%	
353					*TET2*	<5%	
354	51	M	cytosis				NA
355	83	F	concern				NA
356	66	F	cytosis				NA
357	77	F	concern				NA
358	73	M	cytosis				NA
359	78	M	cytosis		*CBL*	22.50 %	Plasma cell neoplasm
359					*TET2*	9.70%	
359					*TET2*	7.90%	
359					*TET2*	<5%	
360	42	F	concern				NA
361	50	F	concern				NA
362	52	F	cytosis				NA
363	61	F	concern				NA
364	29	M	concern				NA
365	23	M	cytosis		*KRAS*	1.50%	MPN (NOS)
366	85	M	cytosis				NA
367	28	F	cytosis				NA
368	32	M	concern				NA
369	47	M	concern				NA
370	48	F	cytosis				NA
371	71	F	cytosis				NA
372	86	F	cytopenia		*ETV6*	38.40 %	MDS (clinical)
373	68	F	concern	*JAK2*			MPN (NOS)

374	47	F	concern				NA
375	38	F	concern				NA
376	73	M	cytosis				NA
377	78	M	cytosis	*JAK2*			MPN (PV)
378	52	F	cytosis				NA
379	39	M	cytosis				NA
380	74	F	cytosis				NA
381	88	F	cytosis		*DNMT3A*	7.40%	Clonal hematopoiesis
382	25	F	concern				NA
383	67	M	cytosis				NA
384	88	M	concern		*SF3B1*	17.40 %	Plasma cell neoplasm
385	72	M	cytosis		*DNMT3A*	7.30%	Clonal hematopoiesis
386	73	F	cytosis				NA
387	75	M	cytosis	*BCR::ABL1*			MPN (CML)
388	43	F	concern				NA
389	30	F	concern				NA
390	60	F	concern				NA
391	62	M	concern		*DNMT3A*	<5%	Clonal hematopoiesis
392	66	M	cytopenia				NA
393	58	F	concern				NA
394	53	M	cytosis				NA
395	34	F	concern				NA
396	68	M	concern		*DNMT3A*	9.60%	Clonal hematopoiesis
397	73	F	cytosis				NA
398	57	F	cytosis				NA
399	69	F	cytosis/cytopenia				NA
400	78	F	cytosis				NA
401	24	M	cytopenia				NA
402	83	F	cytosis		*DNMT3A*	40.60 %	MDS (clinical)
403	51	F	cytosis				NA
404	51	F	cytosis	*JAK2*			MPN (ET)
405	71	M	cytosis				NA
406	48	F	cytosis				NA
407	88	M	cytosis				NA
408	36	F	cytosis				NA
409	82	F	cytosis				NA
410	38	M	concern				NA
411	19	M	cytosis				NA
412	63	F	cytosis		*ZRSR2*	<5%	Plasma cell neoplasm
412					*ASXL1*	15.40 %	
413	59	M	concern		*DNMT3A*	<5%	Clonal hematopoiesis

414	45	F	cytosis				NA
415	83	F	concern				NA
416	24	M	cytosis				NA
417	44	F	concern				NA
418	83	F	cytosis				NA
419	40	M	cytosis				NA
420	29	F	cytosis				NA
421	36	M	cytosis				NA
422	23	M	cytosis				NA
423	58	F	concern				NA
424	82	M	concern				NA
425	93	F	cytosis	*JAK2*			MPN (NOS)
426	70	M	concern				NA
427	74	F	cytosis				NA
428	39	F	concern				NA
429	59	F	cytosis	*JAK2*	*DNMT3A*	<5%	MPN (NOS)
430	64	M	cytosis		*DNMT3A*	<5%	NA
431	19	F	cytosis				NA
432	37	F	cytosis				NA
433	37	M	concern				NA
434	73	M	cytosis				NA
435	75	M	cytosis				NA
436	72	F	cytosis	*JAK2*			MPN (NOS)
437	47	M	cytopenia				NA
438	60	F	concern				NA
439	48	F	cytopenia				NA
440	61	M	concern				NA
441	73	F	concern				NA
442	47	M	concern				NA
443	26	F	concern				NA
444	27	F	cytosis				NA
445	79	M	cytosis		*CEBPA*	<5%	MPN/MDS
445					*ASXL1*	9.20%	
445					*CEBPA*	<5%	
445					*CEBPA*	66.70 %	
445					*SMC1A*	93.00 %	
445					*TET2*	46.60 %	
445							
445							
446	35	F	concern				NA
447	54	M	cytosis		*ASXL1*	47.00 %	Clonal hematopoiesis

448	35	F	cytosis				NA
449	77	F	cytosis				NA
450	33	M	concern				NA
451	56	F	concern				NA
452	66	M	concern				NA
453	24	M	concern				NA
454	68	M	cytosis				NA
455	38	M	concern				NA
456	33	M	cytopenia				NA
457	87	M	concern		*TET2*	<5%	Clonal hematopoiesis
458	48	M	concern				NA
459	83	M	concern				NA
460	26	F	cytosis				NA
461	73	F	cytosis				NA
462	66	F	cytosis				NA
463	58	F	cytosis	*JAK2*			MPN (PV)
464	31	F	cytosis				NA
465	78	M	cytosis				NA
466	44	F	concern				NA
467	42	F	concern				NA
468	48	M	MPN	*JAK2*			MPN (MF)
468				*MPL*			
469	32	M	concern				NA
470	55	F	cytosis				NA
471	75	F	cytosis				NA
472	76	F	cytosis	*JAK2*	*ASXL1*	<5%	MPN (ET)
473	69	M	cytosis				NA
474	31	M	cytosis				NA
475	84	M	concern				NA
476	79	F	concern		*DNMT3A*	5.80%	Clonal hematopoiesis
477	44	F	concern				NA
478	21	F	cytosis				NA
479	77	M	concern				NA
480	69	F	cytosis		*TET2*	<5%	Clonal hematopoiesis
481	81	F	cytopenia		*DNMT3A*	22.70 %	MDS (clinical)
482	46	F	cytosis		*TET2*	<5%	Clonal hematopoiesis
483	46	M	cytosis	*JAK2*			MPN (ET)
484	51	M	cytosis				NA
485	41	M	concern				NA
486	80	F	cytopenia		*TET2*	24.10 %	MDS (clinical)
487	54	M	concern		*U2AF1*	<5%	Clonal hematopoiesis
488	57	F	cytosis				NA

489	63	M	concern		*CBL*	<5%	Clonal hematopoiesis
490	31	F	concern				NA
491	36	M	concern				NA
492	61	M	cytosis				NA
493	44	F	cytosis				NA
494	62	M	cytosis				NA
495	80	M	concern		*TET2*	7.20%	Clonal hematopoiesis
495					*PPM1D*	<5%	
496	62	F	cytosis				NA
497	33	F	concern				NA
498	37	M	cytosis				NA
499	64	M	cytosis				NA
500	23	F	cytosis				NA
501	46	F	concern				NA
502	31	F	concern				NA
503	66	F	concern				NA
504	36	F	concern				NA
505	76	M	cytosis	*CALR*			MPN (MF)
506	42	M	cytosis				NA
507	88	F	concern	*JAK2*			MPN (NOS)
508	79	M	concern		*TET2*	<5%	Clonal hematopoiesis
509	62	F	concern				NA
510	56	F	cytosis				NA
511	96	F	cytosis	*JAK2*	*TET2*	<5%	MPN (NOS)
511					*TET2*	25.80%	
511					*TET2*	11.90%	
512	66	M	concern				NA
513	99	F	concern		*ASXL1*	5.40%	Clonal hematopoiesis
514	54	M	cytosis		*TET2*	<5%	Clonal hematopoiesis
515	35	F	cytosis				NA
516	36	M	concern				NA
517	33	F	cytosis				NA
518	49	F	cytosis				NA
519	58	F	cytosis				NA
520	43	M	cytosis				NA
521	79	M	cytosis				NA
522	78	M	concern		*DNMT3A*	<5%	Clonal hematopoiesis
523	56	M	concern				NA
524	29	F	cytosis				NA
525	35	M	cytosis				NA
526	47	F	concern				NA
527	63	M	cytosis/cytopenia	*CALR*	*TET2*	39.90%	MPN (ET)

528	41	M	concern				NA
529	76	F	cytosis				NA
530	41	M	concern				NA
531	98	M	cytosis/cytopeni a		*DNMT3A*	<5%	Clonal hematopoiesis
532	75	M	cytopenia		*TET2*	12.40 %	Lymphoma
533	82	F	cytosis		*TET2*	42.60 %	MPN/MDS
534	47	M	concern				NA
535	33	F	concern				NA
536	53	F	cytosis		*DNMT3A*	<5%	Clonal hematopoiesis
537	73	F	cytosis		*U2AF1*	10.70 %	Clonal hematopoiesis
537					*DNMT3A*	40.10 %	
538	48	M	concern				NA
539	44	M	concern				NA
540	29	F	cytosis				NA
541	62	M	cytopenia		*DNMT3A*	<5%	Plasma cell neoplasm
541					*DNMT3A*	11.20 %	
542	38	M	concern				NA
543	77	M	concern				NA
544	69	F	concern				NA
545	74	F	concern		*KRAS*	35.30 %	MPN (NOS)
546	62	M	concern				NA
547	32	F	concern				NA
548	29	F	cytosis				NA
549	73	F	concern				NA
550	87	M	cytosis/cytopeni a		*U2AF1*	6.60%	MDS (clinical)
550					*SH2B3*	<5%	
551	56	M	cytosis		*DNMT3A*	<5%	Clonal hematopoiesis
552	30	F	concern				NA
553	48	M	cytopenia				NA
554	55	M	concern				NA
555	30	M	cytosis				NA
556	29	F	concern				NA
557	54	M	concern				NA
558	56	M	concern				NA
559	88	M	concern	*BCR::ABL1*			MPN (CML)
560	40	F	cytosis				NA
561	64	M	concern				NA
562	47	F	cytosis				NA
563	96	M	cytosis	*JAK2*	*U2AF1*	39.50 %	MPN (NOS)

564	77	F	cytosis		*DNMT3A*	<5%	Clonal hematopoiesis
565	88	F	concern		*TET2*	23.50 %	Clonal hematopoiesis
566	43	F	concern				NA
567	33	F	cytosis				NA
568	46	F	cytosis				NA
569	67	F	concern				NA
570	88	M	cytosis	*JAK2*	*SF3B1*	38.70 %	MPN (NOS)
570					*DNMT3A*	36.20 %	
571	88	F	concern	*JAK2*			MPN (NOS)
572	27	F	concern				NA
573	75	M	cytopenia		*DNMT3A*	6.40%	Clonal hematopoiesis
574	53	F	cytosis		*DNMT3A*	6.50%	Clonal hematopoiesis
575	67	F	cytosis		*DNMT3A*	5.30%	Clonal hematopoiesis
576	74	F	concern		*KRAS*	38.80 %	MPN (NOS)
577	52	M	concern				NA
578	69	M	concern				NA
579	78	F	MPN	*JAK2*	*TET2*	9.70%	MPN (MF)
579					*TET2*	<5%	
580	67	M	concern				NA
581	83	F	cytosis	*JAK2*			MPN (NOS)
582	51	F	MPN	*CALR*			MPN (ET)
583	39	F	concern				NA
584	77	F	concern		*DNMT3A*	<5%	Clonal hematopoiesis
584					*DNMT3A*	<5%	
585	76	M	cytosis		*SF3B1*	25.30 %	MPN (PV)
586	70	M	cytosis				NA
587	70	F	cytosis				NA
588	50	F	cytosis				NA
589	73	F	cytosis				NA
590	51	F	cytosis				NA
591	70	F	concern				NA
592	79	M	concern				NA
593	68	M	cytosis	*JAK2*			MPN (PV)
594	35	M	cytosis				NA
595	62	F	cytosis				NA
596	51	F	cytosis				NA
597	74	M	cytosis				NA
598	81	F	concern				NA
599	76	M	cytosis				NA
600	83	F	cytosis		*TP53*	<5%	Clonal hematopoiesis
601	52	F	concern				

602	63	M	concern				NA
603	59	F	concern				NA
604	44	F	concern				NA
605	70	M	cytosis				NA
606	50	M	concern				NA
607	66	F	concern				NA
608	60	M	cytosis				NA
609	53	F	cytosis		*DNMT3A*	<5%	Clonal hematopoiesis
610	51	F	cytosis				NA
611	36	F	concern				NA
612	81	F	concern				NA
613	77	F	concern		*DNMT3A*	<5%	Clonal hematopoiesis
614	72	M	cytosis				NA
615	73	F	concern				NA
616	83	M	concern				NA
617	27	F	concern				NA
618	54	M	concern				NA
619	30	F	cytosis				NA
620	45	M	cytosis		*SH2B3*	49.10 %	Germline origin
621	62	M	cytosis		*DNMT3A*	<5%	Clonal hematopoiesis
622	74	M	concern				NA
623	44	M	concern				NA
624	44	F	cytosis				NA
625	59	F	cytosis				NA
626	55	M	concern				NA
627	43	M	cytosis	*BCR::ABL1*			MPN (CML)
628	62	F	concern				NA
629	67	M	concern				NA
630	54	M	cytosis				NA
631	46	M	cytosis				NA
632	25	F	cytosis	*JAK2*			MPN (NOS)
633	25	F	concern				NA
634	41	F	cytosis				NA
635	78	M	cytosis				NA
636	66	F	cytosis	*JAK2*	*DNMT3A*	5.00%	MPN (PV)
636				*JAK2*			
637	48	M	cytosis	*BCR::ABL1*			MPN (CML)
638	53	M	concern				NA
639	49	M	cytosis				NA
640	41	F	cytosis				NA
641	63	M	cytosis				NA
642	87	F	concern				NA
643	27	F	cytosis				NA

644	87	F	concern				NA
645	29	F	cytosis				NA
646	75	M	concern				NA
647	40	F	MPN	*CALR*			MPN (ET)
648	60	F	concern				NA
649	54	M	concern				NA
650	43	F	cytosis	*JAK2*			MPN (ET)
651	34	F	cytosis				NA
652	51	M	concern				NA
653	42	F	cytosis				NA
654	61	F	cytosis				NA
655	40	F	concern				NA
656	29	M	cytosis				NA
657	44	M	concern				NA
658	49	F	cytosis				NA
659	60	F	cytosis				NA
660	39	F	cytosis				NA
661	50	M	concern		*DNMT3A*	18.10 %	MDS (clinical)
662	72	F	concern				NA
663	48	M	cytosis				NA
664	63	M	cytosis				NA
665	50	M	cytosis				NA
666	72	M	cytosis				NA
667	34	M	cytosis				NA
668	72	M	concern	*JAK2*	*DNMT3A*	19.00 %	MPN (NOS)
668							
669	27	F	concern				NA
670	64	F	concern	*JAK2*	*TET2*	47.80 %	MPN (PV)
670							
671	62	F	cytosis				NA
672	33	F	cytosis				NA
673	52	M	cytosis				NA
674	66	M	cytosis				NA
675	78	F	cytosis				NA
676	62	F	cytosis		*DNMT3A*	<5%	Clonal hematopoiesis
677	32	M	cytosis				NA
678	72	F	concern				NA
679	90	M	concern		*TET2*	43.60 %	MPN/MDS
679					*ASXL1*	37.20 %	
679					*KRAS*	<5%	
679					*RUNX1*	<5%	

679					*SRSF2*	27.20 %	
679					*TET2*	32.10 %	
680	42	M	concern				NA
681	57	M	concern				NA
682	37	M	cytosis				NA
683	48	F	concern				NA
684	30	F	cytosis				NA
685	31	M	concern				NA
686	80	M	concern				NA
687	74	F	cytosis				NA
688	52	F	concern				NA
689	73	M	cytosis				NA
690	55	F	cytosis				NA
691	73	M	cytosis				NA
692	74	F	cytosis				NA
693	64	F	cytosis				NA
694	41	M	concern				NA
695	48	F	cytosis				NA
696	42	M	cytosis				NA
697	45	F	concern				NA
698	89	F	cytopenia		*DNMT3A*	9.70%	Plasma cell neoplasm
699	42	F	cytosis				NA
700	30	M	cytosis				NA
701	37	M	concern				NA
702	58	M	concern				NA
703	50	F	concern				NA
704	72	M	cytosis				NA
705	74	F	cytosis				NA
706	56	M	cytosis/cytopenia				NA
707	60	M	cytosis				NA
708	26	F	cytosis				NA
709	58	M	cytosis/cytopenia				NA
710	51	F	MPN	*CALR*			MPN (ET)
711	80	F	concern				NA
712	63	M	cytosis				NA
713	87	M	concern				NA
714	65	M	concern				NA
715	31	M	cytosis				NA
716	52	M	cytosis	*JAK2*	*TET2*	<5%	MPN (NOS)
717	54	F	concern				NA
718	41	F	cytosis				NA

719	85	M	MPN	*JAK2*	*TET2*	<5%	MPN (ET)
720	40	M	concern				NA
721	69	M	cytosis				NA
722	73	M	cytosis				NA
723	56	F	cytosis				NA
724	68	M	concern		*TP53*	6.20%	MDS (clinical)
724					*CBL*	1.40%	
724					*TET2*	2.30%	
725	32	M	concern				NA
726	33	F	cytosis				NA
727	72	M	cytopenia				NA
728	26	F	cytosis				NA
729	42	M	cytosis				NA
730	86	F	concern		*DNMT3A*	<5%	MDS (clinical)
730					*CBL*	<5%	
730					*CBL*	10.20 %	
730					*TP53*	7.40%	
731	48	M	MPN	*JAK2*			MPN (MF)
731				*MPL*			
732	30	F	cytosis				NA
733	38	F	concern				NA
734	83	M	cytosis	*JAK2*	*SRSF2*	6.10%	MPN (ET)
735	67	M	cytosis		*DNMT3A*	<5%	NA
735					*DNMT3A*	<5%	
736	47	M	concern				NA
737	50	F	concern				NA
738	46	F	cytosis	*MPL*	*DNMT3A*	<5%	MPN (ET)
739	64	F	cytosis				NA
740	69	M	cytosis				Lymphoma
741	40	F	concern				NA
742	37	F	concern				NA
743	70	M	cytopenia				NA
744	59	F	concern				NA
745	69	F	concern				NA
746	45	F	cytosis				NA
747	54	F	cytosis				NA
748	47	F	concern				NA
749	63	M	concern				NA
750	35	M	cytosis		*SH2B3*	<5%	MPN (NOS)
751	57	M	cytosis				NA
752	82	F	cytosis				NA
753	54	F	concern				NA
754	31	F	concern				NA

755	31	M	cytosis				NA
756	20	M	concern				NA
757	58	M	cytosis				NA
758	70	M	concern				NA
759	40	F	cytopenia				NA
760	52	M	concern				NA
761	55	M	concern				NA
762	56	M	cytosis				NA
763	45	F	cytosis/cytopenia				NA
764	53	M	concern				NA
765	55	M	cytosis/cytopenia				NA
766	29	M	cytosis				NA
767	25	F	cytosis				NA
768	59	M	cytosis	*CALR*	*RUNX1*	86.20%	MPN (ET)
769	81	M	cytosis		*SF3B1*	<5%	Clonal hematopoiesis
769					*PPM1D*	<5%	
770	76	M	cytosis				NA
771	49	M	concern				NA
772	91	F	cytopenia		*SF3B1*	36.40%	MDS (clinical)
772					*SRSF2*	<5%	
772					*TET2*	20.50%	
772					*TET2*	9.90%	
772					*TET2*	5.80%	
773	19	F	concern				NA
774	33	F	cytosis				NA
775	76	F	cytosis	*BCR::ABL1*			MPN (CML)
776	84	F	cytosis				Lymphoma
777	87	M	cytosis/cytopenia	*JAK2*			MPN (NOS)
778	69	F	concern				NA
779	45	F	concern				NA
780	61	F	concern				NA
781	65	M	cytopenia				NA
782	44	M	concern				NA
783	75	M	concern		*TP53*	<5%	Clonal hematopoiesis
784	79	F	concern		*DNMT3A*	<5%	Clonal hematopoiesis
785	72	M	cytosis	*JAK2*			MPN (ET)
786	48	M	cytosis				NA
787	69	M	cytosis	*JAK2*			MPN (NOS)
788	74	F	cytopenia				NA
789	61	F	concern				NA

790	57	F	cytosis				NA
791	53	F	cytosis				NA
792	61	F	cytosis/cytopenia		*DNMT3A*	<5%	Clonal hematopoiesis
793	73	F	concern				NA
794	75	F	cytosis/cytopenia				NA
795	33	F	concern				NA
796	46	M	MPN	*JAK2*			MPN (NOS)
797	76	F	MPN	*JAK2*			MPN (NOS)
798	42	M	concern	*JAK2*	*ASXL1*	<5%	MPN (NOS)
799	55	M	cytosis/cytopenia				Plasma cell neoplasm
800	63	F	cytosis	*CALR*			MPN (ET)
801	41	F	concern				NA
802	65	M	concern				NA
803	68	M	cytosis		*DNMT3A*	4.90%	Lymphoma
803							
804	27	M	concern				NA
805	49	M	cytopenia				NA
806	41	M	cytosis		*TP53*	42.10%	NA
807	54	M	cytosis				NA
808	84	M	cytopenia		*TET2*	35.90%	MDS (clinical)
808					*SRSF2*	32.50%	
809	24	F	concern				NA
810	83	M	concern				NA
811	37	F	cytosis				Histiocytic neoplasm
812	59	M	concern				NA
813	17	M	cytosis				NA
814	36	F	cytosis/cytopenia				NA
815	64	F	concern				Plasma cell neoplasm
816	72	M	concern				NA
817	92	M	cytosis/cytopenia				Lymphoma
818	67	M	cytopenia				NA
819	49	M	concern				NA
820	67	F	cytosis				NA
821	72	M	cytosis		*DNMT3A*	<5%	Clonal hematopoiesis
822	90	F	cytopenia		*IDH2*	23.90%	MDS (clinical)
822					*ASXL1*	16.50%	
822					*NF1*		
822					*STAG2*	22.90%	

823	63	F	concern				NA
824	58	F	cytosis				NA
825	70	F	concern				NA
826	68	F	cytosis/cytopeni a				NA
827	70	M	cytosis	*CALR*			MPN (NOS)
828	72	F	cytosis				NA
829	68	F	cytosis				NA
830	64	M	concern				NA
831	81	F	concern		*TET2*	<5%	Clonal hematopoiesis
832	34	F	concern		*BCOR*	6.40%	Clonal hematopoiesis
833	92	M	cytopenia		*DNMT3A*	17.60 %	MDS (clinical)
834	75	M	cytosis		*TET2*	<5%	Clonal hematopoiesis
835	89	F	cytopenia		*SF3B1*	26.20 %	MDS (clinical)
836	61	M	concern				NA
837	28	F	concern				NA
838	85	F	cytosis				NA
839	52	M	concern		*SH2B3*	99.80 %	Germline origin
840	65	M	cytosis	*JAK2*			MPN (PV)
841	65	F	concern				NA
842	63	F	concern				NA
843	38	F	cytopenia				NA
844	71	F	concern				NA
845	39	M	cytosis	*JAK2*			MPN (ET)
846	82	F	concern				NA
847	65	F	cytosis				NA
848	54	M	cytosis				NA
849	53	M	concern		*DNMT3A*	<5%	Clonal hematopoiesis
850	80	M	cytopenia				NA
851	76	M	cytosis		*TET2*	<5%	MPN (NOS)
851					*ASXL1*	17.20 %	
851					*NRAS*	<5%	
852	66	M	cytosis				NA
853	59	F	MPN	*JAK2*			MPN (PV)
854	75	F	concern		*TET2*	<5%	Clonal hematopoiesis
855	77	M	cytosis	*JAK2*	*TET2*	13.90 %	MPN (ET)
855					*SF3B1*	<5%	
855					*SRSF2*	<5%	
856	60	M	cytosis				NA
857	55	F	cytosis				NA
858	66	F	concern		*TET2*	<5%	Clonal hematopoiesis

859	52	M	cytosis				NA
860	48	F	concern		*DNMT3A*	<5%	Clonal hematopoiesis
861	77	M	cytopenia		*DNMT3A*	<5%	Clonal hematopoiesis
862	62	F	cytosis				NA
863	80	F	cytosis	*JAK2*			MPN (NOS)
864	64	F	cytosis				NA
865	57	M	cytosis				NA
866	73	F	cytosis		*DNMT3A*	<5%	Clonal hematopoiesis
867	46	F	cytosis				NA
868	70	M	concern				NA
869	83	F	cytosis	*JAK2*	*TET2*	29.60 %	MPN (ET)
870	24	F	cytosis				NA
871	71	M	cytosis				NA
872	97	M	cytopenia				NA
873	30	F	cytopenia				NA
874	75	M	cytopenia				NA
875	26	F	cytosis	*BCR::ABL1*			MPN (CML)
876	56	M	concern				NA
877	45	F	concern				NA
878	61	M	cytosis				NA
879	72	F	cytopenia				NA
880	82	F	cytosis		*MYD88*	20.50 %	Lymphoma
881	67	M	cytopenia				NA
882	52	F	cytosis				NA
883	40	F	concern				NA
884	86	F	cytosis	*CALR*			MPN (NOS)
884				*CALR*			
884				*JAK2*			MPN (ET)
885	40	F	cytosis				NA
886	61	F	cytosis				NA
887	62	M	cytosis				NA
888	76	M	cytosis				NA
889	77	F	cytosis/cytopenia				NA
890	63	F	concern				NA
891	54	F	cytosis				NA
892	80	M	cytopenia		*ASXL1*	49.10 %	MDS
892					*KRAS*	41.80 %	
892					*SRSF2*	34.20 %	
892					*TET2*		
893	62	F	concern				NA

894	34	M	cytopenia				NA
895	62	F	cytopenia				Plasma cell neoplasm
896	89	M	cytosis				NA
897	50	F	cytopenia				NA
898	65	M	cytosis				NA
899	50	M	concern				NA
900	33	F	cytosis	*JAK2*			MPN (ET)
901	67	M	cytosis				NA
902	73	F	cytopenia		*PPM1D*	<5%	Clonal hematopoiesis
903	22	M	cytopenia				Bone marrow failure (aplastic anemia/MDS)
904	27	F	cytosis				NA
905	38	F	concern				NA
906	70	F	concern				NA
907	37	M	cytopenia				NA
908	31	F	cytosis				NA
909	40	M	cytosis/cytopenia				NA
910	69	F	cytosis				NA
911	73	M	cytosis				NA
912	84	M	cytosis/cytopenia				NA
913	69	M	cytosis/cytopenia				NA
914	77	M	cytopenia	*CALR*	*ASXL1*	15.70 %	MPN (ET)
914					*KRAS*	<5%	
915	69	F	cytosis				NA
916	77	M	cytosis				NA
917	48	M	cytosis				NA
918	34	F	cytosis				NA
919	63	M	cytopenia				NA
920	32	M	concern				NA
921	55	F	cytosis				NA
922	22	M	concern				NA
923	34	F	concern				NA
924	82	M	cytopenia				NA
925	75	F	cytosis		*SF3B1*	<5%	Clonal hematopoiesis
926	59	M	cytosis				NA
927	52	F	concern				NA
928	57	F	cytosis		*DNMT3A*	17.30 %	Clonal hematopoiesis
929	46	F	cytosis	*CALR*			MPN (MF)
930	56	M	cytosis				NA
931	91	M	cytopenia				NA
932	55	M	concern				NA

933	24	M	cytosis				NA
934	63	M	MPN		*DNMT3A*	<5%	Clonal hematopoiesis
935	59	F	cytosis	*JAK2*	*DNMT3A*	<5%	MPN (NOS)
936	50	M	concern				NA
937	35	F	cytosis/cytopeni a				NA
938	25	F	cytosis				NA
939	44	F	cytosis				NA
940	50	F	cytosis				NA
941	48	F	cytosis				NA
942	61	M	cytosis		*DNMT3A*	<5%	Clonal hematopoiesis
943	45	F	concern				NA
944	59	M	cytosis				NA
945	83	F	cytosis/cytopeni a	*JAK2*	*SF3B1*	35.10 %	MPN (ET)
945				*MPL*			
946	83	F	cytosis				NA
947	88	F	concern				NA
948	20	M	cytosis				NA
949	34	M	cytosis				NA
950	69	M	cytosis/cytopeni a				NA
951	34	M	concern				NA
952	54	M	concern				NA
953	51	M	cytopenia				NA
954	69	M	concern				NA
955	86	M	cytopenia		*TET2*	<5%	Clonal hematopoiesis
955					*SF3B1*	<5%	
955					*SF3B1*	6.80%	
956	45	M	concern				NA
957	54	M	cytosis				NA
958	29	M	concern				NA
959	39	F	cytosis				NA
960	82	F	concern		*U2AF1*	21.20 %	Plasma cell neoplasm
961	58	F	cytosis				NA
961	68	M					NA
962	71	M	concern				NA
963	49	F	cytosis				NA
964	73	M	concern				NA
965	Unknow n	F	cytosis/cytopeni a				NA
966	77	F	concern				NA
967	45	F	cytosis				NA
968	40	M	cytosis				NA
969	24	M	cytopenia				Clonal hematopoiesis

970	75	M	cytopenia		*DNMT3A*	10.00 %	
970					*DNMT3A*	<5%	
970					*DNMT3A*	<5%	
970					*TET2*	<5%	NA
971	66	F	cytosis				NA
972	39	F	concern				Clonal hematopoiesis
973	78	M	concern		*PRPF8*	<5%	NA
974	31	F	concern				NA
975	52	M	cytosis				NA
976	31	F	cytosis				Clonal hematopoiesis
977	67	M	cytosis		*SF3B1*	5.00%	NA
978	76	M	cytosis				MPN (PV)
979	57	M	cytopenia	*JAK2*			NA
980	58	M	concern				NA
981	65	M	cytosis				Hereditary hematologic predisposition
982	72	F	cytopenia	*MPL*			Clonal hematopoiesis
983	80	F	concern		*PPM1D*		NA
984	43	F	concern				NA
985	44	M	cytosis				MPN (CML)
986	71	M	cytosis	*BCR::ABL1*			MPN (ET)
987	86	M	concern	*JAK2*	*TET2*	38.80 %	
987				*MPL*	*TET2*	38.50 %	MPN (ET)
988	60	F	cytosis	*CALR*			NA
989	29	F	cytosis				NA
990	77	M	cytosis				NA
991	34	M	concern				NA
992	62	M	concern				NA
993	84	F	cytopenia				NA
994	80	M	cytosis				MPN (PV)
995	78	F	cytosis/cytopenia	*JAK2*	*ASXL1*	34.20 %	
995					*DNMT3A*	<5%	NA
996	55	F	concern				MPN (MF)
997	48	M	MPN	*MPL*			
997				*JAK2*			NA
998	49	M	cytosis				NA
999	29	F	cytosis				NA
1000	68	M	cytopenia				NA
1001	92	F	concern				Hereditary hematologic predisposition
1002	72	M	concern		*RB1*	36.00 %	
1002					*TET2*		Clonal hematopoiesis

1003	53	M	cytosis				Lymphoma
1004	63	M	concern		*MYD88*	25.70 %	NA
1005	49	M	cytosis				NA
1006	49	M	cytosis				NA
1007	37	F	cytosis				NA
1008	37	F	cytosis				NA
1009	63	F	cytosis				NA
1010	41	F	cytosis				NA
1011	69	F	cytosis				MPN (ET)
1012	76	F	cytosis	*CALR*			NA
1013	42	M	concern				NA
1014	31	M	cytosis				Clonal hematopoiesis
1015	79	F	cytosis		*TP53*	9.90%	NA
1016	86	M	cytosis				NA
1017	75	M	cytosis				NA
1018	85	M	cytosis				Clonal hematopoiesis
1019	64	F	concern		*DNMT3A*	<5%	NA
1020	64	M	concern				NA
1021	48	M	cytosis				NA
1022	86	F	concern				NA
1023	41	F	cytosis				NA
1024	71	M	cytosis				NA
1025	43	M	cytosis				NA
1026	72	M	concern				NA
1027	92	M	cytosis				NA
1028	48	F	cytosis				NA
1029	59	M	cytosis				NA
1030	52	F	cytosis				NA
1031	38	F	cytosis				NA
1032	48	F	concern				NA
1033	63	M	cytosis				NA
1034	71	M	cytosis				NA
1035	41	M	cytosis				Clonal hematopoiesis
1036	28	F	cytosis		*ASXL1*	7.80%	NA
1037	40	M	concern				NA
1038	20	M	concern				NA
1039	76	M	cytosis				NA
1040	58	M	cytosis				NA
1041	59	F	concern				NA
1042	23	F	cytosis				NA
1043	78	M	cytosis				Clonal hematopoiesis
1044	82	M	concern		*TP53*	<5%	MPN (ET)
1045	76	F	cytosis	*JAK2*	*SF3B1*	8.00%	NA

1046	23	F	cytosis				Clonal hematopoiesis
1047	79	M	concern		*SF3B1*	<5%	NA
1048	65	M	concern				Clonal hematopoiesis
1049	74	M	cytosis		*DNMT3A*	6.50%	NA
1050	41	M	cytosis				MPN (ET)
1051	77	M	MPN	*JAK2*	*NF1*	<5%	NA
1052	75	F	concern				MPN (CML)
1053	73	M	cytosis	*BCR::ABL1*	*DNMT3A*	7.70%	NA
1054	81	M	concern				NA
1055	71	F	concern				NA
1056	38	M	concern				Clonal hematopoiesis
1057	75	M	concern		*ASXL1*	<5%	
1057					*SF3B1*	9.10%	
1057					*TET2*	<5%	NA
1058	71	F	cytosis				NA
1059	60	M	cytosis				NA
1060	16	M	concern				Clonal hematopoiesis
1061	36	F	concern				NA
1062	27	F	concern				NA
1063	38	F	concern				NA
1064	54	F	cytosis				NA
1065	25	F	cytosis				NA
1066	38	F	cytosis				NA
1067	36	M	concern				MPN (CML)
1068	34	F	cytosis	*BCR::ABL1*			NA
1069	78	F	cytosis				NA
1070	34	M	cytosis				MPN (PV)
1071	79	M	MPN	*JAK2*	*TET2*	15.90 %	
1071					*TET2*	26.00 %	MPN (ET)
1072	90	F	cytosis	*MPL*			NA
1073	58	M	concern				NA
1074	44	M	concern				NA
1075	55	M	cytosis				NA
1076	47	M	cytosis				Lymphoma
1077	63	M	cytosis		*MYD88*	7.20%	NA
1078	65	F	cytosis				NA
1079	69	F	cytosis				NA
1080	31	F	cytosis				Clonal hematopoiesis
1081	67	F	cytosis		*TP53*	<5%	NA
1082	35	M	cytosis				NA
1083	37	F	cytosis				NA
1084	23	F	cytosis				NA

1085	57	M	cytosis				MPN (NOS)
1086	57	F	concern		*SH2B3*	9.00%	NA
1087	22	F	concern				Clonal hematopoiesis
1088	65	F	concern		*PRPF8*	<5%	NA
1089	43	M	cytosis				NA
1090	75	F	cytosis				NA
1091	23	F	cytosis				Clonal hematopoiesis
1092	57	M	concern		*TET2*	<5%	
1092					*TET2*	5.60%	NA
1093	35	M	cytosis				NA
1094	38	M	concern				MPN (ET)
1095	37	M	MPN	*JAK2*			MPN (NOS)
1096	74	F	cytosis/cytopenia		*TET2*	<5%	
1096					*ASXL1*	34.00%	NA
1096					*KRAS*	26.10%	
1097	69	M	concern				MPN (MF)
1098	48	M	cytopenia	*JAK2*			
1098				*MPL*			MPN (NOS)
1099	68	M	concern	*JAK2*			NA
1100	40	M	cytosis				Clonal hematopoiesis
1101	62	F	cytosis		*DNMT3A*	<5%	MPN (NOS)
1102	84	M	concern	*JAK2*			NA
1103	34	M	concern				Lymphoma
1104	74	M	concern		*DNMT3A*	<5%	
1104					*DNMT3A*	10.50%	
1104					*DNMT3A*	11.20%	
1104					*TET2*	12.50%	MPN (NOS)
1105	86	F	cytosis	*JAK2*			NA
1106	52	F	concern				Clonal hematopoiesis
1107	70	F	cytosis		*DNMT3A*	19.40%	NA
1108	69	M	concern				NA
1109	51	M	concern				NA
1110	47	M	cytosis				NA
1111	34	F	concern				NA
1112	71	M	concern				MPN (ET)
1113	97	F	concern	*JAK2*	*TET2*	13.30%	
1113					*PPM1D*	<5%	NA
1114	40	M	concern				NA
1115	36	F	cytosis				NA

1116	44	F	cytosis				NA
1117	46	F	cytosis				MPN (ET)
1118	40	F	cytosis	*CALR*			NA
1119	73	M	concern				NA
1120	65	M	concern				NA
1121	40	M	concern				NA
1122	42	F	concern				NA
1123	86	M	cytosis				MPN (PV)
1124	78	M	cytosis	*JAK2*	*TET2*	5.50%	NA
1125	58	F	cytosis				NA
1126	71	F	cytosis				Plasma cell neoplasm
1127	79	F	concern		*MYD88*	<5%	NA
1128	61	M	concern				Clonal hematopoiesis
1129	81	F	cytopenia		*DNMT3A*	<5%	NA
1130	55	M	cytosis				MPN (PV)
1131	49	F	MPN	*JAK2*	*DNMT3A*	9.90%	NA
1132	86	M	cytosis				NA
1133	70	M	concern				NA
1134	90	M	concern				MPN (ET)
1135	84	F	cytosis	*JAK2*	*TP53*	14.10 %	NA
1136	46	F	cytosis				NA
1137	31	M	concern				NA
1138	54	M	cytosis				Clonal hematopoiesis
1139	66	F	concern		*TET2*	<5%	NA
1140	63	M	cytosis				NA
1141	19	M	cytosis				NA
1142	44	M	cytosis				NA
1143	68	F	concern				MPN (ET)
1144	71	M	cytosis	*CALR*	*DNMT3A*	<5%	NA
1145	67	M	concern				NA
1146	62	F	concern				NA
1147	63	F	cytosis				NA
1148	72	F	cytosis				MPN (ET)
1149	58	M	cytosis	*JAK2*	*U2AF1*	<5%	NA
1150	72	M	cytosis				NA
1151	71	F	concern				NA
1152	38	F	concern				NA
1153	56	M	concern				NA
1154	62	M	cytosis				NA
1155	23	F	cytosis				NA
1156	81	F	concern				MDS (clinical)
1157	67	M	concern		*DNMT3A*	28.90 %	NA

1158	30	M	concern				NA
1159	49	F	cytosis				NA
1160	61	M	concern				NA
1161	76	F	concern				MPN (PV)
1162	63	F	MPN	*JAK2*			Clonal hematopoiesis
1163	77	F	concern		*DNMT3A*	<5%	NA
1164	70	M	cytosis				NA
1165	44	M	cytosis				NA
1166	35	M	cytosis				NA
1167	69	M	concern				NA
1168	71	F	cytosis				NA
1169	68	M	cytosis				MPN (ET)
1170	40	F	MPN	*CALR*			NA
1171	58	M	cytosis				NA
1172	45	F	concern				NA
1173	50	F	concern				NA
1174	45	F	concern				NA
1175	81	M	concern				NA
1176	32	M	concern				NA
1177	22	F	concern				Clonal hematopoiesis
1178	68	F	cytosis		*TP53*	<5%	NA
1179	62	M	cytosis				Clonal hematopoiesis
1180	95	F	concern		*DNMT3A*	<5%	
1180					*DNMT3A*	<5%	NA
1181	43	F	concern				NA
1182	67	M	concern				MPN (NOS)
1183	46	M	cytosis	*JAK2*			NA
1184	56	M	cytosis				NA
1185	24	M	concern				NA
1186	77	F	concern				NA
1187	53	M	cytosis				NA
1188	77	M	concern				Clonal hematopoiesis
1189	65	M	concern		*TET2*	<5%	Clonal hematopoiesis
1190	87	M	concern		*TET2*	7.50%	NA
1191	49	M	cytosis				NA
1192	35	M	cytosis				NA
1193	57	F	concern				NA
1194	63	F	concern				NA
1195	40	M	cytosis				NA
1196	54	M	cytosis				Clonal hematopoiesis
1197	61	F	cytosis		*DNMT3A*	<5%	MPN (CML)
1198	73	M	cytosis	*BCR::ABL1*			NA
1199	75	M	cytosis				NA

1200	72	F	cytopenia				NA
1201	69	M	cytosis				NA
1202	60	F	concern				NA
1203	52	M	concern				NA
1204	35	F	concern				NA
1205	41	F	cytosis				MPN (PV)
1206	87	F	MPN	*JAK2*	*TP53*	<5%	
1206					*DNMT3A*	15.20 %	
1206					*DNMT3A*	11.00 %	NA
1207	68	M	concern				Plasma cell neoplasm
1208	88	M	cytosis		*SF3B1*	11.50 %	NA
1209	36	M	MPN	*BCR::ABL1*	*RUNX1*	74.80 %	MPN (CML)
1209					*ABL1*	24.20 %	
1209					*ABL1*	<5%	

Table S2. SH2B3 variants. Variants are reported according to HGVS nomenclature at the coding DNA (HGVSc) and protein (HGVSp) levels. The table includes variant classification (pathogenic, likely pathogenic, or variant of uncertain significance [VUS]), variant allele frequency (VAF), and predicted molecular consequence (e.g., missense, frameshift, or splice-site alteration).

Patient No.	Age	Domain Affected	HGVSc	HGVSp	Annotation	VAF	Consequence
92	44	SH2-partial	c.1228_1229insG	p.I410Sfs*46	Pathogenic	2.83%	frameshift_variant
99	35	SH2	c.1198G>A	p.E400K	Likely pathogenic *	46.45%	missense_variant
353	76	SH2-partial	c.1238_1245del	p.H413Pfs*40	Pathogenic	2.71%	frameshift_variant&splice_region_variant
367	28	SH2	c.1198G>A	p.E400K	Likely pathogenic *	45.27%	missense_variant
1086	57	SH2-entirely	c.1021+1G>A		Pathogenic	9.04%	splice_donor_variant
478	21	PH	c.706G>A	p.V236M	VUS	51.37%	missense_variant
550	87	SH2	c.1175G>A	p.R392Q	Pathogenic	3.15%	missense_variant
620	45	SH2	c.1183G>A	p.E395K	Pathogenic	49.07%	missense_variant
750	35	SH2-entirely	c.1045dup	p.D349Gfs*36	Pathogenic	4.01%	frameshift_variant
798	42	SH2	c.1226G>C	p.G409A	VUS	51.65%	missense_variant
839	52	SH2	c.1183G>A	p.E395K	Pathogenic	99.80%	missense_variant (Biallelic loss)

* This variant was classified as likely pathogenic based on published disease associations, its location within the functional SH2 domain of SH2B3, and its classification as likely oncogenic in OncoKB; however, ClinVar currently classifies the variant as a variant of uncertain significance (VUS).
